# Adult-Onset Still’s Disease With Severe Hyperferritinemia and the Asian Salmon-Pink Rash: A Case Report

**DOI:** 10.7759/cureus.26257

**Published:** 2022-06-23

**Authors:** Toshiki Fukunaga, Ryuichi Ohta, Fumiko Yamane, Chiaki Sano

**Affiliations:** 1 Family Medicine, Faculty of Medicine, Shimane University, Izumo, JPN; 2 Community Care, Unnan City Hospital, Unnan, JPN; 3 Community Medicine Management, Faculty of Medicine, Shimane University, Izumo, JPN

**Keywords:** salmon-pink rash, tocilizumab, relapse, steroid tapering, fever of unknown origin, hyperferritinemia, adult-onset still’s disease, rural hospital, general medicine

## Abstract

Adult-onset Still's disease (AOSD) is a systemic inflammatory disease characterized by rash, arthritis, and persistent spiking fever. The diagnosis and treatment of AOSD are challenging due to the lack of specific diagnostic criteria and little evidence of effective treatments. Here, we reported a case of an 18-year-old woman with a fever of unknown origin (FUO), evanescent rash (without the typical “salmon-pink” color), and systemic lymphadenopathy. Laboratory tests at hospital admission revealed marked hyperferritinemia of 12,100 ng/mL. AOSD was subsequently suspected. Additional anti-nuclear-antibody analysis for differential diagnosis was negative. The initiation treatment with high-dose prednisolone, tapered to half every week, was immediately started. The symptoms temporarily improved but relapsed during the tapering period. The prednisolone dose was increased again, and tocilizumab was introduced. Symptom remission and prednisolone dose reduction were subsequently achieved. Therefore, a medication tapering schedule and treatment replacement to inhibit the pathophysiology of AOSD need to be carefully considered. While a ferritin test is useful to diagnose AOSD based on the presence of FUO, there are AOSD patients without hyperferritinemia. Additionally, AOSD rash on Asian skin may not present with the typical “salmon-pink” color.

## Introduction

Adult-onset Still's disease (AOSD) is a rare systemic autoinflammatory disease with an annual incidence of 0.16 cases per 100,000 people, characterized by evanescent rash, arthritis, and spiking fever. AOSD often presents with hyperferritinemia, with 70% of the patients showing markedly elevated blood ferritin levels (>3,000 ng/mL). However, this clinical finding is not specific to AOSD [1]. Since diseases exhibiting hyperferritinemia include collagen diseases, infections, and malignant tumors, the exclusion of these conditions is essential to the AOSD diagnosis [2]. The innate immune system, which causes elevated expression of inflammatory cytokines in AOSD patients, is implicated in the pathophysiology of this illness [3]. Serum levels of interleukin (IL)-1 and IL-6 are reportedly correlated with the severity of AOSD symptoms, and cytokine inhibitors, such as anakinra and tocilizumab, are effective treatments for AOSD. Tumor necrosis factor-alpha (TNF-α) expression also increases in AOSD, at a modest magnitude compared to other collagen diseases. However, TNF-α inhibitors such as infliximab have been effective in treating AOSD with chronic arthritis. While the cause of AOSD is considered to be a combination of genetic predisposition and preceding infection, no specific genetic background or infectious microorganisms have been identified [4]. The familial onset of AOSD is also rare. As such, there is little evidence supporting the diagnosis and treatment of AOSD, which are currently decided by the judgment of each clinician.

For AOSD treatment, steroids, non-steroidal anti-inflammatory drugs (NSAIDs), disease-modifying anti-rheumatic drugs (DMARDs), and anakinra are used to induce remission of inflammatory symptoms. Steroids are widely used as first-line treatment drugs for AOSD. Many studies have reported on the efficacy of steroids in AOSD, with a variable response to the dose and AOSD types [4]. The effectiveness of DMARDs. The effectiveness of DMARDs used in rheumatoid arthritis has been reported for AOSD. These medications are also used to replace steroids. Methotrexate shows a 60% response rate for the treatment of AOSD, regardless of the patient's steroid sensitivity [4]. Tocilizumab is an anti-IL-6 antibody with a 60% treatment response rate in AOSD [5]. Anakinra is an IL-1 inhibitor with a 70-90% response rate for the treatment of AOSD alone [6]. While NSAIDs are sometimes used before AOSD diagnosis, their response rate is 16% [4].

AOSD is classified into three types according to the course of the disease and different symptom patterns [7]. The monophasic type has only one active disease period, with systemic symptoms such as fever, rash, serositis, and hepatosplenomegaly, and often results in complete remission within a year. The intermittent type is characterized by the intermittent onset of systemic symptoms, and complete remission can be expected within a few weeks to two years [7]. Symptoms of the intermittent type are similar to those of the monophasic type, and the symptoms become less severe with each recurrence. The chronic type is characterized by persistent symptoms, with predominantly chronic arthritis. Unlike other collagen diseases, complete remission can be expected as the ultimate treatment goal of the monophasic and intermittent types of AOSD. Therefore, the following treatment strategies can be considered according to different AOSD types. The goal of treatment in monophasic AOSD is medication tapering to a level as low as possible and eventual medication termination. A similar treatment strategy is applied to intermittent AOSD, with the additional increase in medication dosage at the time of disease relapse. Chronic AOSD requires continuous treatment. Medication replacement is also considered to avoid long-term steroid use in all types of AOSD. In particular, anakinra and tocilizumab, which inhibit the inflammatory cytokines IL-1 and IL-6, respectively, are reported to have a high steroid-tapering effect [5,6].

Here, we describe a case with a marked hyperferritinemia and fever of unknown origin (FUO) in whom the diagnosis of AOSD was considered and immediate treatment was initiated. While the disease symptoms relapsed during steroid treatment, medication replacement with tocilizumab maintained symptom remission and steroid dose reduction.

## Case presentation

An 18-year-old female patient without any notable family or medical history came to our hospital with the chief complaint of persistent fever, appetite loss, and bilateral neck lymphadenopathy with consultation from a primary care physician. She had recently graduated from high school, and the patient started working and training for a driver’s license. She did not have any travel history or sick contact. Approximately one month before hospital admission, the patient experienced left neck discomfort. Three weeks later, she visited a doctor for a complaint of swollen left cervical lymph nodes and fever. Due to her fever, persistent fatigue, and increased findings of inflammation, she was referred to another doctor and was then treated with anti-inflammatory agents. She was referred to our hospital because of recurrent fever, general malaise, and generalized lymphadenopathy. Further investigations revealed an elevated level of C-reactive protein, an increased erythrocyte sedimentation rate (ESR), and an abnormally high level of ferritin (measured after sample dilution) (Table 1).

**Table 1:** Laboratory findings during the course ESR: erythrocyte sedimentation rate; CRP: C-reactive protein; AST: aspartate aminotransferase; ALT: alanine aminotransferase

	Hospitalization					Outpatient		Re-hospitalization		
	Day 1	Day 2	Day 3	Day 7	Day 11	Day 16	Day 23	Day 26	Day 28	Day 31
Leukocyte count (10^3^/µL)	8.2	8.1	9.5	8.6	10.5	12.2	9.7	7.9	5.7	10.2
Neutrophil (%)	78.8	79.7	78.2		73.3	84.7	85.9	92.8	62.6	73.2
Platelet (10^4^/µL)	18.5	16.7	14.2	17.8	26.9	29.8	16.0	10.3	9.2	19.0
ESR (mm)	39	-	-	-	-	16	21	21	-	-
CRP (mg/dL)	7.56	5.55	2.2	0.55	0.11	0.10	0.20	1.32	-	0.13
AST (IU/L)	125	110	69	73	36	31	38	86	41	29
ALT (IU/L)	51	46	39	140	114	71	60	84	56	51
Ferritin (ng/mL)	12100.0	16663.0		2281.9	390.9	158.7	156	677.5	-	160.4

**Figure 1:**
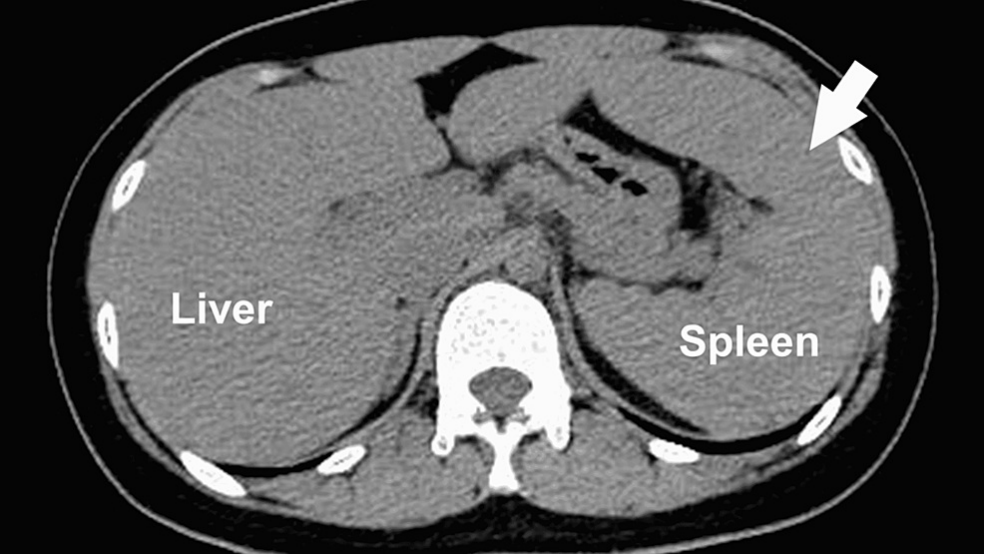
Computer tomography revealing marked splenomegaly (arrow)

**Figure 2:**
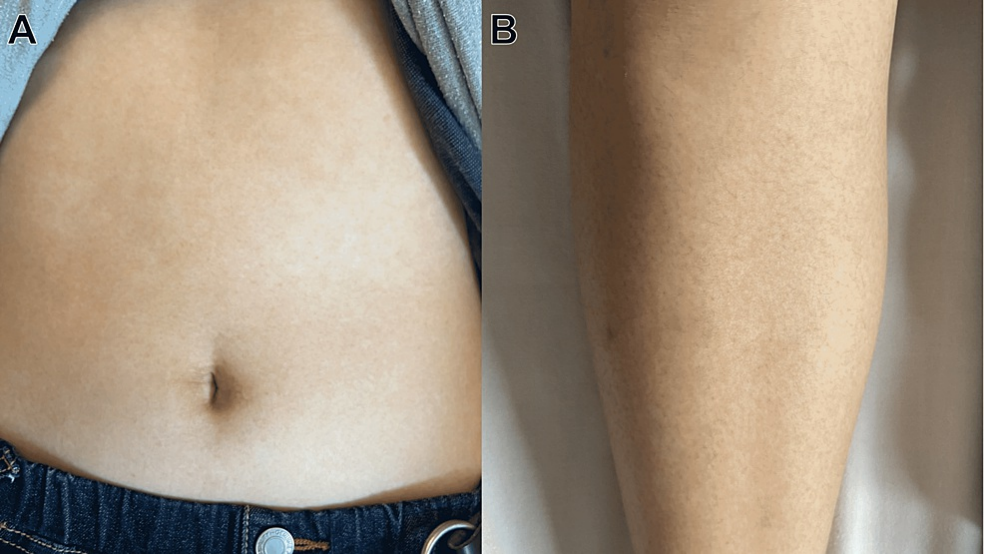
Evanescent painless skin rash on the trunk (A) and leg (B), which doesn’t look like so called “salmon-pink”

Computed tomography showed marked splenomegaly (Figure 1). The investigation of her skin confirmed a rash on her lower extremities and trunk that did not resemble the “salmon-pink” color on Asian skin (Figure 2).

The laboratory tests showed negative autoantibodies and no hematological abnormalities, excluding the diagnosis of other collagen and hematological diseases. We diagnosed the patient with AOSD based on the Yamaguchi criteria. The treatment with prednisolone for AOSD at 50 mg/day (1 mg/kg/day) was initiated. Subsequently, the symptoms improved and the prednisolone dose was tapered by half every week. Her blood test on the 11th day after admission improved her inflammation-related findings, including ferritin levels, and she was discharged on the 14th day. Steroid tapering was continued during the outpatient period and sulfamethoxazole-trimethoprim (ST) combination was prescribed to prevent Pneumocystis pneumonia. However, two days after discharge, the patient came back to our hospital due to symptom recurrence. The prednisolone dose was increased to 20 mg/day. On the ninth day after discharge, her symptoms improved.

**Figure 3:**
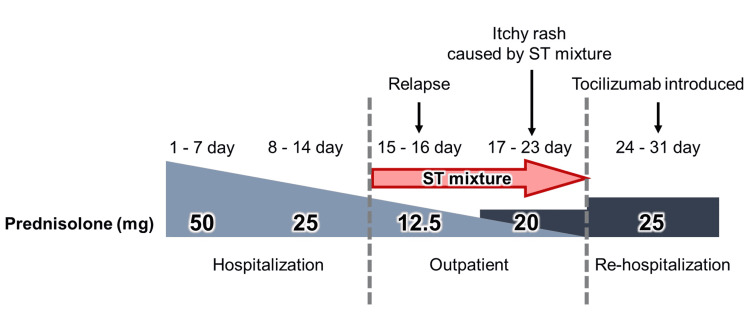
The clinical course of the case ST: sulfamethoxazole-trimethoprim

On the 11th day after discharge, the patient revisited our hospital due to an itchy rash. Since this symptom was considered a side effect of the ST mixture, this treatment was discontinued. A blood test during this hospital visit showed an increase in inflammatory findings, including ferritin, and a decrease in platelet count. Therefore, given the worsening of her AOSD symptoms, the patient was re-hospitalized and a dose increment of prednisolone (up to 25 mg/day) was initiated. The worsening of AOSD symptoms and ST mixture-related rash might have been caused by stress from the patient's busy lifestyle. After readmission, symptom remission was achieved. A blood test on the 6th day of readmission revealed negative inflammatory findings. Tocilizumab was introduced to avoid the long-term use of steroids. Afterward, her symptom remission was maintained and prednisolone dose could be reduced to 10 mg/day (Figure 3).

## Discussion

The patient was diagnosed with AOSD with marked hyperferritinemia, initially treated with steroids with subsequent introduction of tocilizumab. In this case, the following exemplary points were demonstrated. First, treatment should be started immediately for AOSD with hyperferritinemia exceeding 10,000 ng/mL. Second, steroid dose should be reduced by medication replacement, particularly with inflammatory cytokine inhibitors. In addition, the AOSD rash may not present with the “salmon-pink” color on Asian skin.

AOSD diagnosis is often difficult depending on the clinical context and disease. Due to the lack of specific diagnostic findings and the rarity of the illness, it might require one to four months from onset to diagnosis [2]. To date, there are no definitive diagnostic criteria for AOSD, and the diagnosis primarily depends on the judgment of the clinician. The Yamaguchi criteria (Table 2) and Fautrel criteria (Table 3) are known as AOSD classification criteria.

**Table 2:** Yamaguchi criteria, in which for AOSD diagnosis, patients should meet more than five criteria, among which at least two should be major criteria

Four major criteria	Minor criteria
Fever ≥39°C for 1 week	Sore throat
Arthralgia or arthritis for 2 weeks	Lymphadenopathy
Evanescent rash	Hepatosplenomegaly
Leukocytosis ≥10,000/mm^3 ^and neutrophil ≥80%	Liver dysfunction on examination
-	Negative antinuclear antibody

**Table 3:** Fautrel criteria, in which for AOSD diagnosis, patients should meet “four or more major criteria” or three major criteria and two minor criteria

Major criteria	Minor criteria
Spiking fever ≥39°C	Maculopapular rash
Arthralgia	Leukocytes >10,000/mm^3^
Transient erythema	-
Pharyngitis	-
Polymorphonuclear ≥80%	-
Glycosylated ferritin ≤20%	-

Both criteria demand the exclusion of other autoimmune, infectious and hematological diseases. However, the sensitivity of the Yamaguchi criteria is complicated by the number of differential diagnoses [8]. On the other hand, the Fautrel criteria require analysis of glycosylated ferritin levels, which could not be measured in many medical institutions due to the lack of equipment [9]. While algorithms and scoring systems have been developed in recent years to distinguish AOSD from FUO, it is difficult to consider these methods unless AOSD is suspected [10]. Besides, the “salmon-pink” rash is characteristic of AOSD. While AOSD rash on Caucasian skin is “salmon-pink,” this feature was not present on Asian skin in this case. Therefore, the possible absence of the “salmon-pink” rash feature of AOSD should be considered during diagnosis.

Hyperferritinemia is a useful finding for AOSD diagnosis. In this regard, hyperferritinemia is caused by pre-inflammation or iron overload, and AOSD is a typical disease with hyperferritinemia. A 2021 cohort study of patients with hyperferritinemia showed that ferritin levels in AOSD patients were significantly higher than those in other diseases [2]. Additionally, 15 of the 17 patients with hyperferritinemia > 10,000 ng/mL had AOSD. The receiver-operating-characteristic analysis in this study suggested that AOSD could be detected with a sensitivity of 85.71% and a specificity of 81%, with a ferritin threshold of >1,757 ng/mL. Collectively, these findings suggested that serum ferritin levels may contribute to the detection of AOSD in patients with hyperferritinemia.

Collaboration between different medical institutions in rural areas is essential for AOSD diagnosis. Since this case lacked characteristic findings of AOSD, the patient had to visit three medical institutions. Based on previous physician assessments and treatments, we were able to confirm hyperferritinemia in our hospital, start steroid treatment immediately, and then perform tests such as autoantibody analysis for diagnosis by exclusion. As a result, our early intervention was successful and the symptoms showed a transient remission, resulting in early restoration of the patient's quality of life. Therefore, clinical information of patients with FUO should be effectively shared during the diagnosis to facilitate timely analysis of serum ferritin levels. As such, AOSD could be suspected in the presence of severe hyperferritinemia.

AOSD treatment choices depend on its clinical course. This condition is currently treated by steroids, NSAIDs, DMARDs, anakinra, or other agents. However, clinical research on AOSD is difficult due to its rarity, and the 2017 edition of the Adult-onset Still's Disease Practice Guidelines in Japan refrained from recommending first-line medications due to the absence of efficacy data [11]. Since there were no randomized clinical trials directly comparing the effectiveness of different drugs for AOSD as of 2022, treatment is conducted empirically. Prednisolone is commonly used as the first-line treatment for AOSD. Prednisolone has no contraindications other than hypersensitivity. The efficacy of its combined use with desmopressin for AOSD has also been reported. On the other hand, since there are many side effects associated with steroids, avoidance of long-term administration is desirable. Therefore, the goal of steroid treatment for AOSD is to control symptoms and eventually terminate or reduce steroid dosage as much as possible. However, there is no recommended dosing protocol for steroids and the treatment strategy is decided by the judgment of each clinician.

In this case, steroid-tapering therapy with prednisolone was performed. When treating AOSD steroids, starting with a high dose (prednisolone 0.8-1.0 mg/kg/day) increases the likelihood of discontinuation of the medication [12]. Therefore, the prednisolone dose was started at 50 mg/day (1.0 mg/kg/day) and reduced by half each week. In the third week, with a prednisolone dose of 12.5 mg/day, AOSD symptoms relapsed. The dose of prednisolone was then increased to 20 mg/day and the symptoms improved again. These findings suggested that a long dose reduction time might be required in this case.

In this case, the AOSD symptoms recurred along with the ST mixture-induced rash. Abnormalities in the innate immune system due to AOSD may have triggered ST mixture hypersensitivity. Additionally, at the time of relapse, the patient experienced a busy lifestyle and was under stress. Therefore, healthy life is necessary for AOSD patients even in the presence of symptom remission [12].

Tocilizumab, an anti-IL-6 antibody, is a type of biological DMARDs. A 2020 cohort study suggested that tocilizumab is more effective for treatment-resistant AOSD and helpful to reduce steroid dosage and prevent disease relapse [5]. Tocilizumab adherence is easy to maintain as it can be taken once every two to four weeks. However, since this medication has a strong immunosuppressive effect, careful follow-up in severe infections should be considered.

Anakinra, an IL-1 inhibitor, has also been reported to be effective against AOSD [5]. Similar to tocilizumab, anakinra helps to reduce steroid dosage. However, it has a short half-life and requires daily subcutaneous administration [6]. Considering the balance between treatment adherence and infectious disease control, anakinra can be used instead of tocilizumab. However, anakinra is an unapproved drug in Japan and is difficult to use due to administrative procedures.

In this case, the reasons for symptom relapse may be related to the disease type, treatment method, and lifestyle stress [7]. Since this case predominantly showed systemic presentation, symptom relapse might have occurred as a course of the intermittent AOSD subtype. Additionally, therapeutic drugs could be managed by dose maintenance and dose increase during relapse. 

In this case, the disease exhibited steroid resistance after relapse. Compared to other drugs, tocilizumab has been suggested to be more effective in treating refractory AOSD and reducing steroid dose. Medication replacement with tocilizumab at the time of relapse resulted in symptom remission and steroid dose reduction for this patient. It is necessary to consider the use of tocilizumab for AOSD treatment or steroid dose reduction in patients with symptom relapse. In rural contexts, effective follow-up could be performed with ESR to avoid bacteremia or other infections because the usage of tocilizumab decreases the amount of CRP [13].

## Conclusions

This case shows that hyperferritinemia could be useful for the diagnosis of AOSD, and disease remission was successfully maintained by tocilizumab. AOSD lacks characteristic findings and may take some time to be diagnosed. However, since early intervention could improve AOSD prognosis, a method for early detection of AOSD is needed. Measuring serum ferritin in patients with FUO can be helpful for the early detection and intervention of AOSD. In addition, while steroids are one of the important initial therapeutic agents for AOSD treatment, avoidance of long-term use is necessary due to their side effects. Furthermore, since AOSD patients with in-treatment relapse are more likely to be treatment-resistant, long-term management of AOSD requires a resistance-focused strategy. Steroid replacement with tocilizumab may result in steroid dose reduction and improved long-term prognosis. Finally, AOSD rash does not necessarily present with the “salmon-pink” color on Asian skin.
